# Exploring the Relationship between Attitudes, Risk Perceptions, Fatalistic Beliefs, and Pedestrian Behaviors in China

**DOI:** 10.3390/ijerph18073378

**Published:** 2021-03-24

**Authors:** Mingyu Liu, Jianping Wu, Adnan Yousaf, Linyang Wang, Kezhen Hu, Katherine L. Plant, Rich C. McIlroy, Neville A. Stanton

**Affiliations:** 1 Department of Civil Engineering, Tsinghua University, Beijing 100084, China; my-liu16@mails.tsinghua.edu.cn (M.L.); yousafa10@mails.tsinghua.edu.cn (A.Y.); lywang5185@outlook.com (L.W.); 2 China Academy of Information and Communication Technology, Beijing 100191, China; hukezhen@caict.ac.cn; 3 Human Factors Engineering, Transportation Research Group, University of Southampton, Southampton SO16 7QF, UK; K.Plant@soton.ac.uk (K.L.P.); R.Mcilroy@soton.ac.uk (R.C.M.); N.Stanton@soton.ac.uk (N.A.S.)

**Keywords:** pedestrian behaviors, fatalistic beliefs, traffic safety attitudes, risk perceptions

## Abstract

Road safety has become a worldwide public health concern. Although many factors contribute to collisions, pedestrian behaviors can strongly influence road safety outcomes. This paper presents results of a survey investigating the effects of age, gender, attitudes towards road safety, fatalistic beliefs and risk perceptions on self-reported pedestrian behaviors in a Chinese example. The study was carried out on 543 participants (229 men and 314 women) from 20 provinces across China. Pedestrian behaviors were assessed by four factors: errors, violations, aggressions, and lapses. Younger people reported performing riskier pedestrian behaviors compared to older people. Gender was not an influential factor. Of the factors explored, attitudes towards road safety explained the most amount of variance in self-reported behaviors. Significant additional variance in risky pedestrian behaviors was explained by the addition of fatalistic beliefs. The differences among the effects, and the implications for road safety intervention design, are discussed. In particular, traffic managers can provide road safety education and related training activities to influence pedestrian behaviors positively.

## 1. Introduction

The road traffic system is a complex sociotechnical system including humans, technologies, and environments. With the development of technology, the quality of roads and vehicles has been improved; however, as an integral part of the road traffic system, human behaviors are an influential factor in road traffic collisions. Since the first recorded pedestrian fatality in 1899, more than 300,000 pedestrians have died in traffic crashes in the US alone [1]. According to the Traffic Management Bureau of the Ministry of Public Security (TMBMPS), in 2010 there were 16,281 pedestrian deaths and around 45,000 pedestrian injuries due to traffic collisions in China [2]. The World Health Organization (WHO) estimated that more than half of all traffic collision fatalities are vulnerable road users such as pedestrians, cyclists, and people on motorcycles [3]. Therefore, it is essential to understand pedestrian behaviors in order to improve road safety.

Tools for studying pedestrian behaviors can be summarized into two broad categories [4]. One is ethnological observation in the road environment; this has been argued to be the best way of understanding the effects of the environment on pedestrian behaviors in a specific scenario [5,6,7]. However, it is not possible to collect every type of pedestrian behavior under all possible risky situations through observation. Moreover, it is not appropriate for researchers to put pedestrians into dangerous situations. Thus, in order to study risky behaviors, many researchers have used questionnaires (e.g., [8,9,10,11,12]). This method can provide an insight into pedestrian behaviors, for example, in the exploration of the psychological mechanisms that explain intentional and unintentional risky pedestrian behaviors. A number of researchers have proposed questionnaires for studying pedestrian behaviors. Based on the Driver Behavior Questionnaire (DBQ; [8]), Diaz developed a 16-item Pedestrian Behavior Questionnaire (PBQ) in Chile [9]. The research classified risky pedestrian behaviors into three components: violations (deliberate deviations from rules), errors (deficiency in knowledge of traffic rules) and lapses (inattention or memory failures). In 2004, Elliott and Baughan developed a questionnaire for road behaviors in the United Kingdom, the Adolescent Road User Behavior Questionnaire (ARBQ) [11]. The ARBQ included both cyclist and pedestrian behaviors and it differentiated 21 items into three components: unsafe road crossing, dangerous playing in the road and planned protective behavior. Based on that scale, Granié et al. developed a comprehensive self-report measure to differentiate pedestrian road-using behaviors into violations, errors, lapses, aggressions (aggressive actions towards other road users), and positive behaviors (actions considered as polite, or pro-social), with both a long version (37-item) and a short version (21-item) [4]. This was subsequently validated in a North American [13], and across Bangladesh, China, Kenya, Vietnam, and the UK ([14,15,16]).

The role of demographics in pedestrian behaviors has been examined by a variety of researchers, with a common finding being that males report performing riskier behaviors than females [13,17,18]. That said, some studies have failed to show the effect of gender on pedestrian behaviors [19,20,21]. As for the effect of age, the literature has pointed to a negative correlation between age and risky pedestrian behaviors, such that older pedestrians report performing safer behaviors than younger pedestrians [4,22,23].

Social cognition theories, such as the theory of planned behavior [24], suggest that attitudes predict behavior, something that has been explored in the pedestrian behavior contexts across a number of high-income countries [25]. Papadimitriou et al., for example, studied 19 European countries and showed a strong link between attitudes and pedestrian behaviors [26]. The result suggested that pedestrians with negative attitudes toward safety measures and in-vehicle devices, such as speed-limiting devices, report performing more risky pedestrian behaviors. In Turkey, a middle-income country, a study showed that safer attitudes toward rule violation, risk, and violations were correlated with less risky pedestrian behaviors [27]. The majority of the studies examining the role of pedestrian attitudes in pedestrian behaviors have been undertaken in western countries. Survey conducted by Zhou and Horrey is one exception to this; they examined adolescent pedestrian behaviors in Beijing, showing that attitudes are significant predictors of the behavioral intentions to perform a range of pedestrian behaviors (including road crossing behaviors) [28]. They concluded, however, that more studies are necessary. To the best of authors’ knowledge, only limited research focus on the Chinese adult population. Therefore, it is essential to validate the effect of attitudes when predicting pedestrian behaviors for Chinese people.

In addition to demographic information and attitudes towards road safety, beliefs are another possible correlate of pedestrian behaviors. People with a fatalistic mindset are more likely to explain unexpected life events by factors such as fate, God, luck, chance, just reward and just punishment factors, which are generally considered to be unchangeable. Thus, for people with highly fatalistic beliefs, it may be less important to take precautions or obey traffic rules, following the idea that accidents will occur in the end no matter what has been done [29,30]. This was demonstrated in South Africa, where the more fatalistic participants were, the less they used their seatbelt [31]. Kouabenan carried out research on causal attributions of traffic collisions in The Ivory Coast [32]. The results suggested that fatalistic drivers involved in collisions will admit their powerlessness, while denying their responsibility, and that people with higher degrees of fatalism tend to take more risks than others. We argue, therefore, that fatalistic beliefs could be a potentially important factor for predicting risky pedestrian behaviors. However, few studies have examined the effect of fatalism on road user behaviors, and there is a complete lack of studies examining the role of such beliefs in risky pedestrian behaviors. Thus, in order to fill in the gap, the present study aimed to examine the role of fatalism in predicting pedestrian behaviors.

Finally, risk perception is a measure of the perceived probability of experiencing a negative event (e.g., [33]). According to social cognitive health models, such as the health belief model [34], there is a direct link between risk perception and behavior. If a person perceives a behavior as more risky, they are less likely to perform it. This has been found both among drivers and pedestrians [25,35,36]. However, Lund and Rundmo, in survey work in Norway and Ghana, found that risk perception had only a modest association with risky traffic behaviors in Ghana [37]. Such a relationship has not yet been explored with regards to pedestrian behaviors in China.

The overall aim of this research is to improve pedestrian safety in traffic social technology system, by shedding light on the relationships between demographic factors, attitudes to road safety, fatalistic beliefs, risk perceptions, and self-reported pedestrian behaviors in China. Based on previous work (e.g., [20]), it was hypothesized that gender would not have a significant effect on self-reported pedestrian behaviors in a Chinese sample. It was also hypothesized that safe attitudes towards road safety would explain significant variance in self-reported behaviors, with riskier attitudes linked to riskier self-reported behavior. Following the literature cited above, we hypothesized that those reporting a greater perception of risk would also report performing safer pedestrian behaviors. Finally, following research discussed above [29,30,32], the fourth hypothesis was that fatalistic beliefs would be significantly related to risky pedestrian behaviors, with more fatalistic individuals reporting performing riskier pedestrian behaviors. This study builds on previous published work (see [14,15,16]) that used the same questionnaire data; however, where McIlroy and colleagues made comparisons between six countries (China included), the current research provides a detailed and focused exploration of only the China data, and includes the investigation of risk perceptions, where McIlroy and colleagues’ work did not.

## 2. Materials and Methods

### 2.1. Materials

As aforementioned, the current research focusses on Chinese respondents to a questionnaire that was distributed across six countries (see [14,15,16]). Although previous works were conducted on the same questionnaire across six countries [14,15,16], this study explains China data in detail and investigates the factor of risk perceptions, where previous works did not. The survey instrument consisted of six sections: demographics (11 items), attitudes towards road safety (22 items), fatalistic beliefs (18 items), risk perception on the road (15 items) and pedestrian behaviors (20 items). The scale to measure participants’ attitudes to road safety was developed by [30,38] and was modified for this study. In addition to the 15 questions taken from [38] and four questions from [30], three new items were developed for this study; these related to riding a motorbike without a helmet, riding a bicycle without a helmet, and wearing a seat belt in a car. Fatalistic beliefs or fatalism were defined in different ways in the past years. For example, fatalism was defined as the acceptance of one’s situation in [39], while fatalism was defined as the belief that outcomes are predetermined by external forces in [40]. In this study, to be distinguished with fatalism factor, the block was named fatalistic beliefs, referring to locus of control [16]. To measure fatalistic beliefs, 30 item questionnaire was adopted from [41]; this separated fatalism into five factors, each measured with six items; internality (expectancies of internal control over life), divine control (a strong belief in the influence of a God), luck (luck determines the result of events), helplessness (a pessimistic outlook on life), and general fatalism (events are fixed in advance). On-road risk perception was measured by 15 items taken from the survey described by [42]. To measure pedestrian behaviors, a short version of the Pedestrian Behavior Questionnaire (PBQ) reported by [13] was used. This measured five factors (positive behaviors, errors, violations, aggressions and lapses) with four items each, and with one modification; in China, it is not a violation of law to cross the road with a red pedestrian light, thus, this item was replaced by another one which concerns the non-use of pedestrian footbridges or underpasses. All items measured responses on a five-point Likert scale, from ‘strongly agree’ to ‘strongly disagree’, except the PBQ, which used a six-point scale, from ‘extremely infrequently or never’ to ‘extremely often or always’.

### 2.2. Respondents

**Figure 1:**
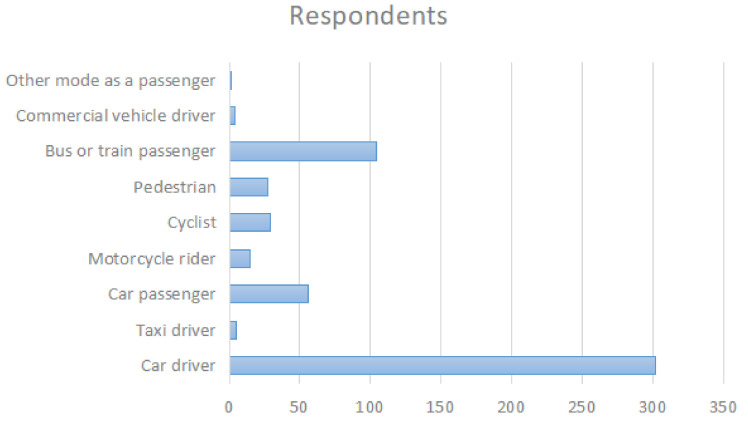
Participants’ most commonly used transport mode.

The survey was conducted via the largest Chinese online survey platform, Wenjuanxing (https://www.wjx.cn/ (Accessed date: 17 January 2021)) and all data were collected from April and September 2018. A link was disseminated through social networks, and through the Wenjuanxing platform itself. The average time to complete the questionnaire was 16 min, and each person was paid 10 Chinese Yen for their participation. A total of 851 road users participated in the study. Wenjuanxing provided a service to recognize conflicting answers of reverse-scaled items and 307 samples were deleted during the procedure. The sample of 543 included responses from 20 provinces, four municipalities, four autonomous regions, and four special administrative regions within China. Age and gender splits for the 543 respondents are shown in Table 1. Among the 543 participants, 26.47% of the respondents reported having been involved in road collision where someone was badly injured, 73.53% did not. The respondents’ most commonly used means of transport was also elicited, the results for which are shown in Figure 1.

## 3. Results

### 3.1. Dimensionality and Reliability of Scales

Statistical analyses in this article were performed using SPSS version 23 (IBM Corp., Armonk, New York, USA). The pedestrian behavior and fatalism sections were taken directly from the literature, with the factors reported in the original work [14,42] retained here. As the attitudes and risk perception sections were compiled from different sources, with some additions unique to the current research, principal component analysis (PCA) was carried out. Cronbach’s Alpha values were calculated to assess internal reliability of the factors used; reliability was considered acceptable if alpha was more than 0.7 (e.g., [43]). The alpha values, means and standard deviations for these factors are shown in Table 2, Table 3, Table 4 and Table 5.

For the attitudes towards road safety section, PCA revealed a single factor to best represent the data (following removal of seven items that had factor loadings lower than 0.4; see [44]). These are presented in Table 2 alongside means and standard deviations. The value of alpha for this scale was 0.81. Higher scores in this section related to safer attitudes towards road safety.

On-road risk perception was best represented by two factors; ‘likelihood of event’ and ‘likelihood of injury’, each of which had good internal reliability, at α = 0.87 and 0.75 respectively. For both risk perception factors, higher scores related to a perception of greater risk.

All five factors of the section on fatalistic beliefs (taken from [41]) were considered to have achieved acceptable internal reliability (measured using Cronbach’s alpha). Although the ‘fatalism’ and ‘divine control’ factors achieved an alpha of below 0.7, they were considered, at 0.69, to be sufficiently close to the 0.7 threshold to merit inclusion. Results associated with these factors should, therefore, be taken with extra caution. Higher scores in this section corresponded to more fatalistic beliefs.

The Pedestrian Behavior Questionnaire ([13]) was split into five factors. The alpha values indicated that all the factors except for Positive Behaviors had acceptable internal reliability (again, at 0.68, the alpha value for Errors was sufficiently high to merit retention of the factor). As such, the Positive Behaviors sub-scale was excluded from all subsequent analyses. Higher scores in this corresponded to riskier pedestrian behaviors.

### 3.2. Regression Analysis

To assess the extent to which demographic factors, attitudes towards road safety, fatalistic beliefs, and risk perceptions explained variance in self-reported pedestrian behaviors in the Chinese sample, four multiple linear regressions models were calculated; one for each of the pedestrian behavior factors. The coefficients for the explanatory variables and the variances explained by the regression models are displayed in Table 6. Before regression analysis, some pre-processing was necessary. Gender and age factors were considered as dummy variables, with males as the reference category. Age factor was divided into four groups: 18–24 years old, 25–34 years old, and over 34 years old; over 34 was the reference group.

**Figure 2:**
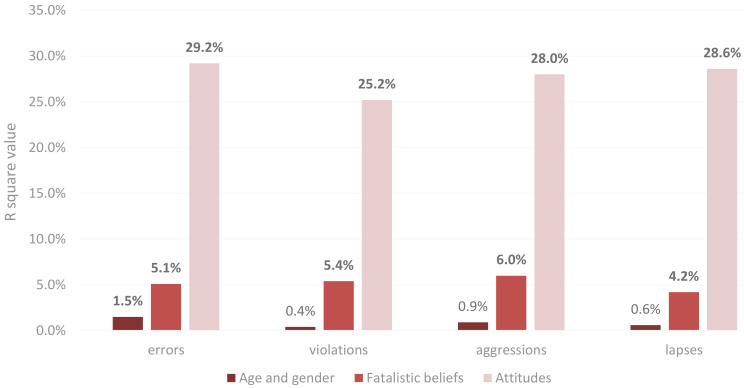
R^2^ values for the prediction of PBQ scores (errors, violations, aggressions and lapses) considering the constructs of age and gender, fatalistic belief and attitudes. Bold numbers indicate statistically significant R^2^ values (*p* > 0.05).

Figure 2 shows the extent to which variance in each PBQ factor was explained by age and gender, fatalistic beliefs, and attitudes (risk perceptions, shown in Table 6, are not included in the figure due to the failure to reach statistical significance). The R^2^ values of age and gender did not achieve significance in the regression models for predicting violations, aggressions and lapses. Although age and gender together were statistically significant predictors of self-reported errors, they only accounted for 1.5% of variance in that factor. The regressions models showed that males and females did not differ significantly in their responses to the four pedestrian behavior factors. There were, however, significant differences between age groups. People in the 25–34 group were found to report performing significantly more pedestrian errors, lapses, and violations than those in the over 34 group. People in 18–24 also reported performing significantly more errors and lapses than those in the older group.

**Figure 3:**
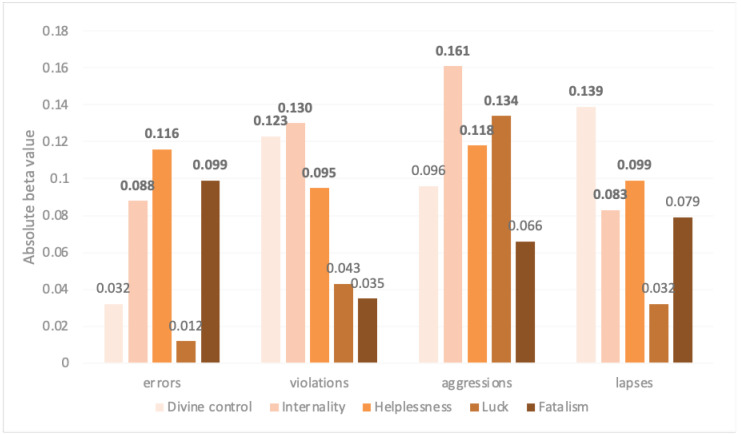
Absolute beta values of fatalistic belief factors (divine control, internality, helplessness, luck and fatalism) in regression models for predicting PBQ scores (errors, violations, aggressions and lapses). The bold numbers indicate statistically significant beta values (*p* > 0.05).

Attitudes towards road safety explained more variance in self-reported pedestrian behavior scores than any of the other factors. This was the case in all regression models; those who reported less safe attitudes also reported performing riskier pedestrian behaviors. Although attitude was the most influential factor, fatalistic beliefs also accounted for a statistically significant additional amount of the variance in the four regression models, over and above age, gender, and attitudes. Figure 3 shows the extent to which each PBQ factor score was explained by the fatalistic belief factors (divine control, internality, helplessness, luck, and fatalism), after age, gender, attitudes, and risk perceptions are held constant. In other words, for each one unit increase in each fatalistic belief factor score, when controlling the other factors, the PBQ score increased or decreased by the amount shown in the figure. For example, the beta value of helplessness was −0.116 when predicting error pedestrian behaviors, which means that the error score decreased 0.116 with one unit increasing in helplessness. Helplessness and internality were important in all four models, although significance levels differed. Divine control was significantly related to violations and lapses. Fatalism was also an important factor when explaining variances in error scores, while luck was significantly related to aggression scores. In all cases, a greater degree of fatalism corresponded to greater reporting of risky pedestrian behaviors. None of the components of risk perception was significantly related to pedestrian behavior scores; none of the beta nor R^2^ values achieved significance in any of the four regression models.

## 4. Discussion

The current study investigated the role of age, gender, road safety attitudes, risk perceptions and fatalistic beliefs in self-reports of risky pedestrian behaviors in a Chinese sample. The results largely supported the hypotheses of this study. In line with previous work in China [19], data showed no significant differences between males and females in the extent to which they reported performing risky pedestrian behaviors. As hypothesized, attitudes towards road safety explained a significant amount of variance in all four of the pedestrian behavior factors, with variance explained ranging from 25.4% (in violations) to 29.5% (in errors). Respondents with more dangerous attitudes to road safety were more likely to report performing riskier behaviors as pedestrians. Fatalistic beliefs were also significantly related to self-reported pedestrian behaviors, though additional variance explained was lower, ranging from 2.8% (for lapses) to 4.6% (for errors). Results showed that risk perception was not significantly related to the self-reported performance of risky pedestrian behaviors; this is in line with the findings from work in Ghana [37], but in contradiction to work undertaken in Norway and Russia [35,36]. 

### 4.1. Age and Gender 

In consideration of the effect of age, findings confirmed previous results that young people are more likely to report performing intentional and unintentional risky pedestrian behaviors. In particular, the result of this study showed that those in the 25–34 age group were significantly more likely to report performing errors (failures of planned actions) and rule violations as pedestrians than those in the over 34 age group. One possible reason may be that unawareness of traffic rules, high energy, and lack of alternatives to walking may lead younger pedestrians to more aggressive and less compliant behaviors [13]. As for the factor of gender, in line with [19,20,21], there were no significant gender differences in self-reported pedestrian behaviors. More traffic safety education should be provided for young people. For example, some videos can be provided for new students at universities. 

### 4.2. Attitudes towards Road Safety 

The results showed a significant effect of attitudes towards road safety on self-reported pedestrian behaviors; this effect was stronger than any other factor, in all the regression models. In line with previous research, we found that people with safer attitudes towards road safety were less likely to report performing risky pedestrian behaviors [28,45]. An alternative interpretation is that attitudes may corresponded to behavior because people adapt their attitudes in a wish to justify their previous actions, not vice versa [46]; this would require further study to clarify. In related work that used the same data as those which are used in the current article, China was the country in which attitudes were most strongly associated with behavior [15]. This would suggest China to be a suitable target for road safety campaigns aimed at improving attitudes towards road safety. For example, the government could oblige companies to provide annual road safety training for employees to improve their attitudes towards road safety, positively influencing their on-road behaviors. Based on the theory of planned behavior (TPB), attitude towards the behavior is an important predictor for individual’s intention, as a performance of given behavior. In this study, these reported risky pedestrian behaviors can be considered as the factor of intention for the TPB. Therefore, attitudes towards road safety was reasonably an important factor for predicting risky pedestrian behaviors.

### 4.3. Risk Perception

Many studies have shown risk perception to be a significant predictor of risky traffic behaviors across a variety of countries (e.g., [25,36]). In this study, however, the risk perception variables did not contribute to a significant percentage of explained variance in any of the pedestrian behavior factors. Results similar to ours have been found in some low-income countries. For example, in research carried out in Ghana, a low-income setting, risk perception was not found to be an important factor in predicting risky road user behaviors [37]. One possible reason is that the questionnaire applied in this study was not designed specifically for China, hence was perhaps unsuitable for the Chinese culture. More effort would need to be made to validate this assumption. It may also be worth questioning the hypothesized causal relationship between risk perception and behavior in general. For instance, [47] have suggested that risk perception is a consequence of behavior, rather than being the cause of it. 

### 4.4. Fatalistic Beliefs

One aim of this study was to investigate whether fatalistic beliefs relate to self-reports of risky pedestrian behaviors. Fatalistic beliefs are akin to locus of control in this study, including divine control, internality, helplessness, luck and fatalism. The regression analyses showed that significant additional variance in risky pedestrian behaviors was indeed explained by the addition of fatalistic beliefs to the model; this was most pronounced for aggressions, and least so for lapses, but was generally comparable in amount across the four behavioral factors. Approximately 5% additional variance in behavior was explained by adding in these factors. Although this is much less than was explained by attitudes, it is more than was explained by age, gender, or risk perceptions. In line with previous works [29,48], people with more fatalistic beliefs reported performing riskier pedestrian behaviors. 

Helplessness, referring to a pessimistic outlook on life, was an important factor that had a statistically significant effect on all four categories of risky pedestrian behaviors. In the traditional culture of China, there is an acceptance that what can be done to change difficult situations is limited, and that the hardship in people’s life is fixed from birth. It is possible that people reporting high levels of helplessness, a feeling common to sufferers of depression, do not care about the potential repercussions of performing risky behaviors. This is lent support from research in the mining industry in China, where it was found that miners who felt helpless against the avoidance of work accidents were more likely to be involved in such accidents [49].

Internality, which defined as expectancies of internal control over important life events, was another factor significantly related to the four types of risky pedestrian behaviors. People who had a more internal locus of control over important life events reported to performing fewer risky behaviors. This concept is closely related to the idea of perceived behavioral control [24]; those who believe they have control over something are more likely to perform a behavior to that end. In a safety context, if an individual believes they have the power to protect themselves, they will do so. Conversely, if they believe safety to be outside of their influence, they may consider it pointless to avoid certain behaviors or actions, and instead accept speed or efficiency goals (e.g., in quickly crossing the road) over safety.

Divine control, which reflects a strong belief in the influence of God in one’s life, was significantly related to violations (deliberate deviations from rules without intention to cause injury or damage) and lapses (involuntary deviations in the action related to a lack of concentration on the task); those who reported a stronger belief in divine influence reported performing more behaviors of these types. The majority (95%) of the sample reported being atheist (80%) or Buddhist (15%), with only 20 individuals (less than 4% of the sample) identifying as either Christian or Muslim, the only theistic religions represented in our sample. As such, this finding should be interpreted cautiously; however, the finding is largely congruent with those reported in other cultures (e.g., [31,32]). Those with a tendency to view events as externally controlled are more likely to violate safety rules. That said, the link between a belief in divine control and a tendency to make inattention and memory errors is not something that has been previously demonstrated or discussed. This would merit further study.

The fatalism factor in this study reflected the core fatalism construct, i.e., a tendency to view all events as inevitable, or fixed in advance [41]. With regards to self-reported pedestrian behaviors, it was significantly related only to the errors factor, referring to deficiency in knowledge of traffic rules in the inferential processes involved in making a decision. This could be related to the theory of destiny in the Chinese culture, whereby one might consider it unnecessary to learn about traffic rules given that outcomes are pre-destined. This would be regardless of any efforts made. This is in accordance with existing research [30,31,32].

Regarding the luck factor, which meant that luck determined the result of events, this was related significantly only to aggressive behaviors. Aggression in this study refers to aggressive interactions with different types of road users [4]. Luck is involved in the interpretation of karma in Buddhism in China [50]. A potential explanation for our finding, therefore, is that people in China with a strong belief in luck have the opinion that good behaviors can help people earn luck in their life in the future. Given that aggressive behaviors could be perceived to represent the opposite of this, it follows that those who subscribe to this belief would consider the performance of aggressive behaviors to potentially result in lower luck in later life (and, as a result, perform fewer of those behaviors). A more detailed treatment of luck, karma, and behavior would be required to shed further light on this issue.

### 4.5. Limitations

The main limitation to this study concerns the sample. All the respondents to the questionnaire completed it online, and most of them had a high level of education, with younger age groups over-represented (compared to the wider population); this will have introduced some bias. Moreover, china is a large country having cities with various developing levels, such as small cities, medium cities and mega cities. Samples were limited regarding the large land area. The second limitation is that only self-report measures were used in this study. The behavior data was not observed pedestrian behaviors, and it is quite possible that some people do not report their risky behaviors to the same extent as they perform them. This limitation can only be solved by collecting actual involvement data and using direct observation. However, this would be challenging, and there would be potentially serious ethical implications involved. A third possible limitation is that we used self-reports of pedestrian behaviors but took general road safety attitudes, many items of which measured attitudes to the use of motorized vehicles. The two things do not match completely, and this might be why the amount of explained variance (i.e., the R^2^ values) was lower than seen in some other behavior-attitude studies. Measuring the relationship between pedestrian-specific attitudes and pedestrian risk behaviors in China would therefore represent a valuable avenue for further research (see [51], for such work in Iran and Pakistan).

## 5. Conclusions

This study investigated the extent to which a variety of factors could explain variance in the self-reported pedestrian behaviors of a Chinese sample. Age was found to be an influential factor, but gender was not. Attitudes towards road safety was the most influential factor in explaining variance in risky pedestrian behaviors, in line with results in other studies. Additionally, Chinese people with more fatalistic beliefs were more likely to report unsafe pedestrian behaviors. In particular, people with a pessimistic outlook on life (i.e., high on feelings of helplessness) reported performing riskier pedestrian behaviors for all four factors investigated. Furthermore, people with an external locus of control, and those reporting a stronger belief in divine influence over one’s life reported performing riskier pedestrian behaviors. Moreover, people who reported a stronger belief in the influence of luck also reported performing fewer aggressive behaviors; this may be influenced by the traditional Chinese interpretation of luck, and its similarity to the concept of karma. Finally, risk perception was not significantly related to self-reported pedestrian behaviors. Results have implications for road safety intervention design, particularly for public education campaigns aimed at influencing the behavior of road users.

## Figures and Tables

**Table 1:** Age and gender characteristics of the samples in China.

	18–24	25–34	Over 34	Total
Male	23 (4%)	113 (21%)	93 (17%)	229 (42%)
Female	60 (11%)	189 (35%)	65 (12%)	314 (58%)

**Table 2:** The alpha values, means and standard deviations for attitudes towards road safety.

Factors	Items	Mean	SD
Attitudes (α = 0.81)	Many traffic rules must be ignored to ensure traffic flow	4.44	0.73
	Traffic rules must be respected regardless of road and weather conditions	4.32	1.12
	It is acceptable to drive when traffic lights shift from yellow to red	4.42	0.93
	It is acceptable to take chances when no other people are involved	4.50	0.67
	Traffic rules are often too complicated to be carried out in practice	3.98	0.88
	If you are a good driver it is acceptable to drive a little faster	3.82	0.87
	When road conditions are good and nobody is around driving at 100 mph (~160 kmh) is ok	4.45	0.78
	I will ride with someone who speeds if that’s the only way to get home at night	4.26	0.77
	I will ride with someone who speeds if others do	4.03	0.79
	I don’t want to risk my life and health by riding with an irresponsible driver	4.44	1.03
	I would never ride with someone I knew has been drinking alcohol	4.49	1.02
	When the road is clear, there is no need to stop at a stop sign	4.08	0.80
	Towards the crest of a hill, a driver should overtake the vehicle in front if they are going faster	4.25	0.86
	It is acceptable to ride on a motorbike without a helmet	4.56	0.72
	You should always wear your seatbelt when travelling in a car	4.66	0.78

**Table 3:** The alpha values, means and standard deviations for on road risk perception.

Factors	Items	Mean	SD
Likelihood of event (α = 0.87)	Head on collision	3.52	1.07
	Vehicle running off the road	3.51	1.03
	Vehicle overturns in the roadway	3.44	1.26
	Collision with a pedestrian	3.60	1.13
	Collision with another vehicle at a road junction	3.75	1.04
	Vehicle explosion following collision	3.26	1.49
Likelihood of injury (α = 0.75)	As a pedestrian	3.60	1.01
	As a rider of a bicycle	3.75	0.87
	As a rider of a motorcycle	4.25	0.85
	As a passenger of a motorcycle or motorised three-wheeler	4.00	0.86
	As a passenger of a car	3.34	0.88

**Table 4:** The alpha values, means and standard deviations for fatalistic beliefs.

Factors	Items	Mean	SD
Fatalism (α = 0.69)	If bad things happen, it is because they were meant to happen	3.88	0.79
	Life is very unpredictable, and there is nothing one can do to change the future	3.06	1.14
	If something bad is going to happen to me, it will happen to me no matter what I do	3.76	0.97
	There is no sense in planning a lot; if something good is going to happen, it will	3.71	0.96
	People die when it is their time to die and there is not much that can be done about it	4.14	0.91
	I have learned that what is going to happen will happen	3.74	0.95
Internality (α = 0.69)	What people get out of life is always due to the amount of effort they put in	3.71	0.98
	What happens to me is a consequence of what I do	3.56	0.98
	I can do almost anything if I really want to do it	3.04	1.12
	What happens to me in the future mostly depends on me	4.02	0.94
	My life is determined by my own actions	4.03	0.90
	I feel that when good things happen, they happen as a result of my own efforts	3.95	0.92
Divine control (α = 0.73)	Everything that happens is part of God’s plan	3.76	0.96
	Everything that happens to a person was planned by God	4.00	0.94
	Whatever happens to me in my life, it is because God wanted it to happen	4.06	0.86
	God controls everything good and bad that happens to a person	4.10	0.89
	God has a plan for each person, and you cannot change His plan	4.00	0.93
	No matter how much effort I invest into doing things, in the end, God’s decision will prevail	4.00	0.92
Luck (α = 0.70)	When good things happen to people, it is because of good luck	3.30	0.95
	When I get what I want, it’s usually because I am lucky	3.70	0.81
	The really good things that happen to me are mostly because of luck	3.38	0.97
	Some people are simply born lucky	2.81	1.15
	How successful people are in their jobs is related to how lucky they are	3.80	0.95
	Luck does not exist	2.79	1.02
Helplessness (α = 0.81)	I feel that nothing I can do will change things	4.14	0.79
	No matter how hard I try, I still cannot succeed in life	4.36	0.76
	I often feel overwhelmed with problems, since I do not have control over solving these problems	3.42	1.05
	Sometimes I fell there is nothing to look forward to in the future	3.86	1.07
	I feel that I do not have any control over the things that happen to me	3.91	0.85
	There is nothing I can do to succeed in life, since one’s level of success is determined when one is born	3.78	0.93

**Table 5:** The alpha values, means and standard deviations for the Pedestrian Behavior Questionnaire (PBQ).

Factors	Items	Mean	SD
Errors (α = 0.68)	I cross between vehicles stopped on the roadway in traffic jams	2.96	1.25
	I cross even if vehicles are coming because I think they will stop for me	2.52	1.25
	I walk on cycling paths when I could walk on the pavement	2.23	1.18
	I run across the street without looking because I am in a hurry	2.11	1.12
Violations (α = 0.81)	I cross diagonally to save time	2.35	1.25
	I cross outside the pedestrian crossing even if there is one (e.g., a crosswalk or zebra crossing) less than 50m away	2.19	1.21
	I avoid using pedestrian bridges or underpasses for convenience, even if one is located nearby	1.97	1.21
	I take passageways forbidden to pedestrians to save time	2.23	1.19
Agreesions(α = 0.89)	I get angry with another road user (pedestrian, driver, cyclist, etc.), and I yell at them	1.99	1.18
	I cross very slowly to annoy a driver	1.67	1.08
	I get angry with another road user (pedestrian, driver, cyclist, etc.), and I make a hand gesture	1.73	1.12
	I have gotten angry with a driver and hit their vehicle	1.60	1.10
Lapses (α = 0.86)	I realize that I have crossed several streets and intersections without paying attention to traffic	1.94	1.14
	I forget to look before crossing because I am thinking about something else	2.14	1.08
	I cross without looking because I am talking with someone	2.17	1.06
	I forget to look before crossing because I want to join someone on the pavement on the other side	2.00	1.14
Positive behaviors (α = 0.54)	I thank a driver who stops to let me cross	2.27	1.11
	When I am accompanied by other pedestrians, I walk in single file on narrow pavements so as not to bother the pedestrians I meet	2.36	1.04
	I walk on the left-hand side of the pavement so as not to bother the pedestrians I meet	2.03	1.00
	I let a car go by, even if I have the right-of-way, if there is no other vehicle behind it	2.83	1.28

**Table 6:** Results of the regression models for pedestrian behaviors.

Blocks	Indicator	Errors			Violations			Aggressions			Lapses		
		Beta	SE	ΔR^2^	Beta	SE	ΔR^2^	Beta	SE	ΔR^2^	Beta	SE	ΔR^2^
1	Gender and age			0.015 *			0.004			0.009			0.006
	Gender	−0.052	0.062		−0.029	0.072		−0.019	0.070		0.011	0.068	
	18–24	0.086 *	0.097		0.050	0.114		−0.053	0.110		0.096 *	0.107	
	25–34	0.172 ***	0.070		0.100 *	0.082		0.045	0.079		0.116 **	0.077	
2	Attitudes	−0.430 ***	0.080	0.292 ***	−0.375 ***	0.093	0.252 ***	−0.401 ***	0.090	0.280 ***	−0.425 ***	0.088	0.286 ***
3	Risk perceptions			0.000			0.001			0.000			0.001
	Likelihood of event	0.005	0.036		−0.046	0.042		−0.016	0.040		−0.001	0.039	
	Likelihood of injury	0.009	0.052		0.014	0.061		0.003	0.059		−0.032	0.057	
4	Fatalistic beliefs			0.051 ***			0.054 ***			0.060 ***			0.042 ***
	Divine control	−0.032	0.064		−0.123 *	0.075		−0.096	0.073		−0.139 **	0.071	
	Internality	−0.088 *	0.056		−0.130 **	0.065		−0.161 ***	0.063		−0.083 *	0.062	
	Helplessness	−0.116 *	0.067		−0.095 *	0.079		−0.118 *	0.076		−0.099 *	0.074	
	Luck	−0.012	0.060		0.043	0.071		0.134 **	0.069		−0.032	0.067	
	Fatalism	−0.099 *	0.067		−0.035	0.078		−0.066	0.076		0.079	0.074	
	Constant		0.358			0.420			0.406			0.395	
Total R^2^				0.358 ***			0.312 ***			0.349 ***			0.335 ***

* *p* < 0.05; ** *p* < 0.01; *** *p* < 0.001. SE: standard error

## Data Availability

The data presented in this study are available on request from the corresponding author.
